# Elevated Fecal pH Indicates a Profound Change in the Breastfed Infant Gut Microbiome Due to Reduction of *Bifidobacterium* over the Past Century

**DOI:** 10.1128/mSphere.00041-18

**Published:** 2018-03-07

**Authors:** Bethany M. Henrick, Andra A. Hutton, Michelle C. Palumbo, Giorgio Casaburi, Ryan D. Mitchell, Mark A. Underwood, Jennifer T. Smilowitz, Steven A. Frese

**Affiliations:** a Evolve BioSystems, Inc., Davis, California, USA; b Foods for Health Institute, University of California, Davis, California, USA; c Department of Pediatrics, UC Davis Children’s Hospital, Sacramento, California, USA; d Department of Food Science and Technology, University of California, Davis, California, USA; e Department of Food Science and Technology, University of Nebraska, Lincoln, Nebraska, USA; The Jackson Laboratory for Genomic Medicine

**Keywords:** *Bifidobacterium*, biochemistry, infant microbiome, microbiome

## Abstract

Historically, *Bifidobacterium* species were reported as abundant in the breastfed infant gut. However, recent studies in resource-rich countries show an increased abundance of taxa regarded as signatures of dysbiosis. It is unclear whether these differences are the product of genetics, geographic factors, or interventions such as formula feeding, antibiotics, and caesarean section. Fecal pH is strongly associated with *Bifidobacterium* abundance; thus, pH could be an indicator of its historical abundance. A review of 14 clinical studies published between 1926 and 2017, representing more than 312 healthy breastfed infants, demonstrated a change in fecal pH from 5.0 to 6.5 (adjusted *r*^2^ = 0.61). This trend of increasing infant fecal pH over the past century is consistent with current reported discrepancies in *Bifidobacterium* species abundance in the gut microbiome in resource-rich countries compared to that in historical reports. Our analysis showed that increased fecal pH and abundance of members of the families *Enterobacteriaceae*, *Clostridiaceae*, *Peptostreptococcaceae*, and *Veillonellaceae* are associated, indicating that loss of highly specialized *Bifidobacterium* species may result in dysbiosis, the implications of which are not yet fully elucidated. Critical assessment of interventions that restore this ecosystem, measured by key parameters such as ecosystem productivity, gut function, and long-term health, are necessary to understand the magnitude of this change in human biology over the past century.

## Abstract (precis)

Historically, *Bifidobacterium* species were reported as abundant in the breastfed infant gut. However, recent studies in resource-rich countries show an increased abundance of taxa regarded as signatures of dysbiosis.

## IMPLICATIONS

There is clear evidence that the infant gut microbiome has important long-term health implications, but changing the gut microbiome is challenging. We recently observed changes in fecal pH resulting from *Bifidobacterium infantis* EVC001 colonization owing to this bacterium’s selective and acidic fermentation of human milk oligosaccharides (HMOs), which was associated with a reduction in taxa that are signatures of dysbiosis. Although remodeling of the gut microbiome in breastfed infants fed *B. infantis* EVC001 improved gut function and ecosystem productivity, questions remain about whether differences in *Bifidobacterium* abundance and species between resource-rich and resource-poor countries are due to host genetics, geography, medical interventions, and/or demographics. Here, we show evidence for an increase in infant fecal pH over the past century, corresponding to an observed reduction of *Bifidobacterium*, the keystone infant gut symbiont. This may have implications for epidemic human immunological dysfunctions as perturbations in microbiota composition can lead to chronic inflammation and immune-mediated diseases.

## EARLY DESCRIPTIONS OF THE INFANT MICROBIOME

In 1913, Logan described the breastfed infant gut microbiome as being an “almost pure culture” of a Gram-positive, acidiphilic “*Bacillus bifidus*” (*Bifidobacterium*) (1). This early microscopic characterization of diet-dependent infant microbiomes is in stark contrast to modern reports from resource-rich countries of unstable and highly diverse microbiomes (2). Recent comparisons of the infant gut microbiome from genetically similar but demographically diverse backgrounds indicated that *Bifidobacterium* was more abundant among infants from resource-poor locations (3), consistent with infants in sub-Saharan Africa and South Asia (4, 5). These differences are also notable at the species level, in that the *Bifidobacterium* in the feces of infants in Gambia and Bangladesh were shown to be predominantly *Bifidobacterium longum* subsp. *infantis* (*B. infantis*), whereas the *Bifidobacterium* species in stool samples from infants in the United States and Europe consisted predominantly of *B. breve* and *B. longum* subsp. *longum* (*B. longum*) (2, 6, 7). Substantial differences in *Bifidobacterium* composition and abundance among populations have led to questions as to whether medical interventions (e.g., caesarean section, antibiotic use) and formula feeding, or geographic and genetic differences alone, results in these differences (2, 6).

## FECAL pH IN BREASTFED INFANTS IS DRIVEN BY *BIFIDOBACTERIUM* ABUNDANCE

**FIG 1:**
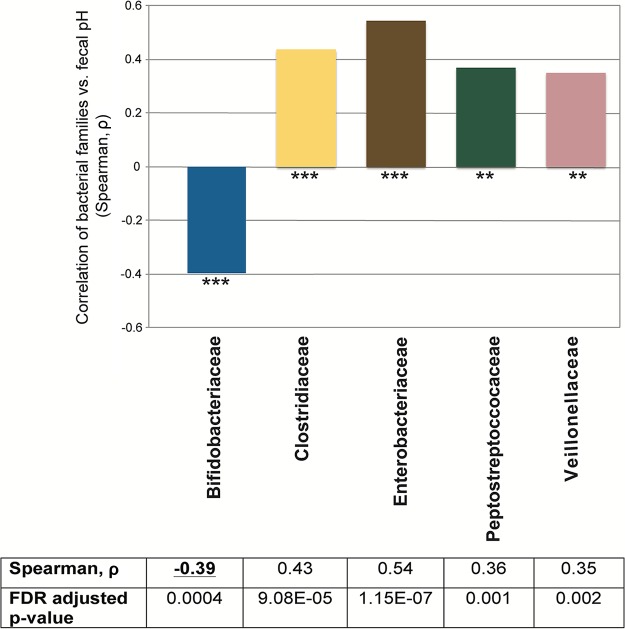
Correlation of bacterial families identified via 16S rRNA marker gene sequencing with fecal pH. Corresponding *P* values were considered statistically significant when they were ≤0.05 with false-discovery rate (FDR) correction. *, *P* < 0.05; **, *P* < 0.01; ***, *P* < 0.001.

Recently, we found that breastfed infants fed *B. infantis* EVC001 developed a stable population of this strain and experienced substantial changes in intestinal biochemistry. Notably, fermentation of HMOs resulted in the increased production of lactate and acetate, which was markedly lower in infants who lacked populations of *Bifidobacterium* or were colonized by other *Bifidobacterium* species. This was concurrent with significantly higher fecal excretion of HMOs than that of infants fed *B. infantis* (7). Using data published by Frese et al. (7), we compared fecal pH measurements with bacterial taxa by using a Spearman correlation. Importantly, only one family was significantly associated with reduced fecal pH, i.e., *Bifidobacteriaceae* (*P* < 0.0004), indicating that while other bacteria can consume HMOs (e.g., *Bacteroidaceae*), only members of the family *Bifidobacteriaceae* convert them to acidic end products with a meaningful effect on fecal pH (Fig. 1). This corroborates previous findings linking infant fecal pH to *Bifidobacteriaceae* abundance (7, 8). This is a critical connection because although other bacteria (e.g., *Lactobacillus*, *Clostridiaceae*, *Lachnospiraceae*, and* Ruminococcaceae*) may produce organic acids during fermentation (e.g., lactate, acetate, butyrate, propionate), they were not significantly associated with the acidic fecal pH in breastfed infants. Infants colonized by *B. infantis* EVC001 had negligible levels of HMOs in their feces and an average fecal pH of 5.15, whereas infants lacking *B. infantis* had 10-fold higher levels of HMOs in their feces and a fecal pH of 5.97 (7). Further, quantitative PCR confirmed the association of low fecal pH with an increased abundance of *Bifidobacterium*, in agreement with another study (8).

## INFANT FECAL pH CHANGES OVER GENERATIONS

Early 1900s reports suggest a rapid reduction in the fecal pH of breastfed infants during the first week after birth (9). Gyorgy and others identified a “bifidus factor,” whose abundance contributed to this reduction in fecal pH and an increase in *Bifidobacterium* in infant feces (10). This “bifidus factor” (now collectively described as HMOs), is selectively consumed by infant-associated *Bifidobacterium*; therefore, pH may be a reliable proxy of the breastfed infant gut microbiome. Infant fecal pH reported over the past century is independent of microbiological methodologies (e.g., microscopic examination versus 16S rRNA gene sequencing); thus, we speculated that historical reports of fecal pH could be used as an indirect measure of *Bifidobacterium* abundance.

**FIG 2:**
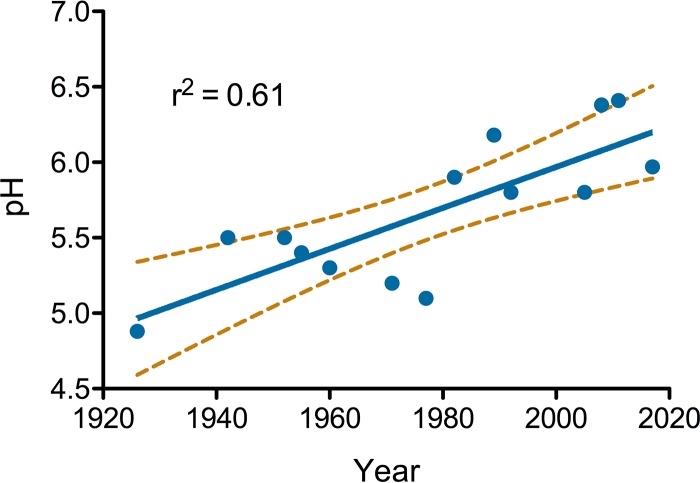
Fecal pH reported in studies along with the average, standard deviation, and numbers of samples measured (where reported) plotted by year of study publication. A linear trend (solid line) and 95% confidence interval (dashed lines) are plotted.

Fourteen peer-reviewed studies published between 1926 and 2017 and reporting 312 measurements from healthy, breastfed infants were found and included. A least-squares linear regression model revealed a strong positive trend with a high association between the publication year and fecal pH (slope = 0.014, adjusted *r*^2^ = 0.61; Fig. 2). These data suggest that the mean fecal pH of breastfed infants has increased from about 5.0 in 1926 to 6.5 in recent years (Table 1). Given our previous finding linking fecal pH to *Bifidobacterium* abundance (7) and reported differences in *Bifidobacterium* abundance across populations today (2, 3, 6, 7), this longitudinal change is consistent with a generational loss of *Bifidobacterium* in developed countries, most notably among infants born after 1980.

**TABLE 1:** Studies examining the fecal pH of healthy, breastfed infants

Study author(s) (country)	Yr	Fecal pH	SD	Sample size	Reference
Eitel (Germany)^a^	1917	4.6–5.6	NR^b^	NR	19
Freudenberg and Heller (Germany)^a^	1921	4.8–5.6	NR	NR	20
Tisdall (Canada)^a^	1924	4.7–5.1	NR	NR	21
Norton (United States)	1926	4.88	0.22	19	9
Uldall (Denmark)	1942	5.5	0.56	17	22
Barbero (United States)	1952	5.5	NR	7	23
Pratt (United States)	1955	5.4	NR	71	24
Nagai (Japan)	1960	5.3	0.25	9	25
Bullen (United Kingdom)	1971	5.2	0.43	10	26
Bullen (United Kingdom)	1977	5.1	NR	13	27
Simhon (United Kingdom)	1982	5.9	NR	17	28
Balmer (United Kingdom)	1989	6.18	0.67	38	29
Ogawa (Argentina)	1992	5.8	0.6	7	30
Knol (Germany)	2005	5.8	NR	21	31
Mohan (Germany)	2008	6.38	0.1	32	32
Holscher (United States)	2011	6.41	0.11	33	33
Matsuki (Japan)^a^	2016	5.9	0.6	15	8
Frese (United States)	2017	5.97	0.57	18	7

a Report excluded for insufficient data.

b NR, not reported.

## FACTORS LEADING TO THIS CHANGE IN INFANT FECAL pH

The absence of *Bifidobacterium* as a keystone symbiont in infants may explain the increase in fecal pH and can be linked to unintended historical and generational consequences of certain interventions that have otherwise significantly improved infant and maternal health. First, a rapid increase in the use of human milk replacers (e.g., evaporated milk and infant formula), which lack the bacterial selectivity of human milk, beginning in the 1920s may have resulted in the inability to foster high levels of specialized infant-associated *Bifidobacterium* in the infant gut among nonbreastfed infants. This may also explain why *B. infantis*, which is highly specialized for the consumption of HMOs, is now exceptionally rare among infants in the United States and Europe, whereas *B. longum* and *B. breve*, which can access mucin glycans and plant carbohydrates (11), remain relatively abundant. Second, increased caesarean section delivery since the 1980s further limits the natural fecal-oral transfer of *Bifidobacterium* from mother to infant associated with vaginal delivery (12). Third, antibiotic use has become increasingly common during labor and many infant-associated species of bifidobacteria are sensitive to antibiotics (13). For example, the use of antibiotics to prevent the transmission of group B *Streptococcus* during delivery and the use of caesarean section as the mode of delivery are both critically important interventions in public health but can alter the acquisition of gut microbes by the infant that begins at birth (13, 14). Together, these barriers may have played a role in the loss of *Bifidobacterium* over time and across generations, which is reflected in a higher fecal pH.

## ARE THERE HEALTH IMPLICATIONS TO THIS CHANGE?

There is clear evidence that the infant gut microbiome has important long-term health implications, and perturbations of the microbiome composition may lead to chronic inflammation (15) and immune-mediated diseases (3, 16–18). These data highlight an increase in infant intestinal dysbiosis (16). Thus, the loss of *Bifidobacterium* and the profound change in the gut environment, as measured by fecal pH, present a compelling explanation for the increased incidence of allergic and autoimmune diseases observed in resource-rich nations. Longitudinal analyses studies comparing the incidence of autoimmune disorders with restored *Bifidobacterium* populations in the infant gut microbiome are essential to establish the role of *Bifidobacterium* in early immune development in the infant gut.
